# Development of Infectious Disease Emergency Response Competencies for Nurses in China: A Delphi Study and an Analytic Hierarchy Process

**DOI:** 10.1155/2023/9952280

**Published:** 2023-05-16

**Authors:** Fengjiao Chen, Li Li, Jiping Li, Hongxia Guo, Xiaoyi Cao, Shu Gong

**Affiliations:** ^1^Department of Hematology, West China Hospital, Sichuan University/West China School of Nursing, Sichuan University, #37 Guo Xue Xiang Street, Chengdu 610041, Sichuan, China; ^2^Intelligence Library Center, West China Hospital, Sichuan University, #37 Guo Xue Xiang Street, Chengdu 610041, Sichuan, China; ^3^Department of Nursing, West China Hospital, Sichuan University/West China School of Nursing, Sichuan University, #37 Guo Xue Xiang Street, Chengdu 610041, Sichuan, China; ^4^West China Hospital, Sichuan University/West China School of Nursing, Sichuan University, #37 Guo Xue Xiang Street, Chengdu 610041, Sichuan, China

## Abstract

**Aim:**

To develop a set of infectious disease emergency response competencies specific to frontline nurses in China.

**Background:**

Nurses play an important role in the infectious disease emergency response. Competency-based training is the cornerstone of the professionalization of disaster rescue, including the infectious disease emergency response. Accordingly, reaching a consensus on a set of core competencies is essential. However, information regarding the competencies needed for nurses in the infectious disease emergency response is limited.

**Methods:**

A literature review and in-depth expert interviews were conducted to establish a draft of competencies, which consisted of 53 items, including 3 first-level index items, 12 second-level index items, and 38 third-level index items. Eighteen experts with the knowledge of infectious disease management and experience with infectious disease emergency rescue from different regions in China were recruited for Delphi consultation. A two-round Delphi survey was conducted via email. Consensus was defined as a mean importance value >4.5 and the coefficient of variation <0.25 among the experts. Finally, the analytic hierarchy process was used to determine the weight of each index on which consensus had been reached.

**Results:**

An index system of infectious disease emergency response competencies for nurses was constructed, including 3 first-level indices (knowledge, attitudes, and skills), 10 second-level indices, and 32 third-level indices. The response rates of the two rounds of the Delphi survey were both 100%, and the authority coefficient of the 18 experts was 0.903. The weighted value of each index was established with a consistency ratio <0.1, demonstrating that skill (0.5396) ranked first among the three first-level indices, followed by knowledge (0.2970) and attitudes (0.1634).

**Conclusion:**

The study developed a consensus on infectious disease emergency response competencies required for nurses in China, which provides guidance for the assessment and training of nurses on infectious disease emergency response. *Implications for Nursing Management*. According to the competency index system, nursing managers could develop effective training programs of infectious disease emergency response competency for nurses and select competent nurses for emergency response to infectious diseases.

## 1. Introduction

In recent decades, emerging infectious diseases have increased due to socioeconomic, environmental, and ecological factors [1, 2]. In general, humans lack natural immunity to emerging infectious diseases; therefore, they may spread rapidly and cause devastating consequences [3]. Correspondingly, the risk of unpredictable infectious disease pandemics is increasing and has become a potential threat to global security. Currently, the entire world is encountering the largest infectious disease emergency, the coronavirus disease 2019 (COVID-19) pandemic. As of November 27, 2022, 637 million cases of COVID-19 and 6.6 million deaths have been reported worldwide [4]. Global health, economies, and social development have been significantly affected.

Although a clear definition of an infectious disease emergency is lacking, it involves two characteristics: infectious disease-related and public health events [5]. Therefore, in this study, we regarded infectious disease emergencies as public health emergencies caused by epidemics or pandemics of infectious diseases, such as severe acute respiratory syndrome (SARS), H1N1, Middle East respiratory syndrome (MERS), Ebola virus disease, and COVID-19. Medical rescue is an essential part of the infectious disease emergency response. Nurses have always played an important role in the response to infectious disease emergencies, including infection prevention, infection control, isolation, containment, and public health [6]. Especially in the absence of effective medical interventions for an emerging infectious disease, nurses play a vital role in caring for patients with fatal infectious diseases [7–9]. For example, during the COVID-19 pandemic in China, approximately 42,000 health care workers nationwide, more than 68% of whom were nurses, were sent to support Hubei Province [10].

Nurses' knowledge and experience are the key to controlling infectious disease pandemics [11]. A group of skilled and competent nurses is essential to adapt to a rapidly changing work environment and provide high-quality care, especially in response to infectious disease emergencies [12]. However, due to the shortage of specialty nurses for infectious disease, most nurses who participate in infectious disease emergency rescue are from noninfectious departments with no prior experience caring for patients with infectious diseases [13]. For example, Labrague and Santos [14] found that 91.4% of the frontline nurses reported that they were not completely prepared to care for COVID-19 patients and highlighted the need to improve nurses' core competencies to better handle infectious disease emergencies. Moreover, frontline nurses are repeatedly exposed to the virus, and providing sufficient training and equipment to protect them is consequently essential [15].

Competence-based emergency nursing staff deployment and training are the cornerstones of the professionalization of disaster rescue, including the infectious disease emergency response [16–18]. Accordingly, reaching a consensus on a set of core competencies is essential. Competence is a complex concept and has been defined as the ability to perform a task with desirable outcomes [19], “functional adequacy and the capacity to integrate knowledge and skills with attitudes and values into the specific contexts of practice” [20], or “the effective application of a combination of knowledge, skill, and judgment demonstrated by an individual in daily practice or job performance” [21]. In this study, the framework of the Chinese registered nurse's competency [22] is adopted for the term nursing competence. According to this framework, nursing competence refers to the integration of knowledge, skills, and attitudes in clinical nursing practice [22]. Therefore, in this study, we define infectious disease emergency response competencies as the integration of knowledge, skills, and attitudes in effective response to an infectious disease emergency for nurses.

To our knowledge, very few studies have demonstrated the specific competencies that are required for nurses in the infectious disease emergency response. Kan et al. [5] developed a competency index system for medical staff in response to infectious disease emergencies. In the index system, only the competencies shared by physicians and nurses were included. However, in the actual situation of infectious disease emergency response, the specific tasks and abilities required for physicians and nurses are quite different. Physicians are mainly responsible for diagnosis and treatment decisions, whereas nurses need to provide specific care activities. Medical diagnosis and treatments are lifesaving but temporary. In contrast, nurses remain at the patient's bedside for long periods and provide the necessary labor-intensive and time-consuming care for the patient's recovery and rehabilitation [23]. Therefore, a scientific and comprehensive competency index system of infectious disease emergency responses for the nurses needs to be developed to guide high-quality training and effective nursing staff deployment during an epidemic. Thus, this study presents the following guiding question: what knowledge, skills, and attitudes are needed by frontline nurses in response to infectious disease emergencies? This study aims to develop a set of infectious disease emergency response competencies specific to frontline nurses in China.

## 2. Methods

This study took place from July 2020 to January 2021. We conducted a modified Delphi consultation to construct the index system and then used the analytic hierarchy process to determine the weight of each index.

### 2.1. Establishment of the Research Team

We established a research team that included 2 nursing education experts, 1 nursing management expert, 1 clinical nursing expert, and 1 nursing graduate student. The responsibility of the research team included developing expert consultation questionnaires, recruiting and contacting experts, and summarizing and analyzing expert opinions.

### 2.2. Construction of the Index System

#### 2.2.1. Construction of the Index Draft and Consultation Questionnaire

In this modified Delphi consultation, we used an initial draft of the index system to replace the traditional first-round Delphi survey. The index draft was constructed through theory analysis, literature review, and a face-to-face in-depth expert interview. First, we adopted the framework of the Chinese registered nurse's competency [22] as the theoretical framework and divided “competencies” into three first-level elements: knowledge, attitudes, and skills. Second, a literature review and expert interviews were conducted, and the data were classified into any dimension of knowledge, attitude, and skills. Eight experts with infectious disease rescue experience participated in 30–60 minutes of one-on-one interviews. In the interviews, the participants were asked approximately 6 questions as follows: According to your experiences and opinions, (1) What roles are played by frontline nurses in response to infectious disease emergencies? (2) What tasks should be undertaken by frontline nurses in response to infectious disease emergencies? (3) What knowledge is needed for frontline nurses in response to infectious disease emergencies? why? (4) What skills are needed for frontline nurses in response to infectious disease emergencies? why? (5) What attitudes and personal attributes are needed for frontline nurses in response to infectious disease emergencies? why? and (6) Is there anything else you would like to share with me? Subsequently, based on the literature [5, 22, 24, 25] and the results of expert interviews, we listed the second-level index and the third-level index related to knowledge, attitudes, and skills. Furthermore, each item was discussed by the research team, and modifications were made where necessary. Finally, an initial draft of 53 indices, including 3 first-level indices, 12 second-level indices, and 38 third-level index items, was developed. The consultation questionnaire consisted of four parts: (1) questionnaire introduction, including the research background, consultation purpose, completion instructions, and contact information for the research team; (2) main body of the questionnaire, including a five-point Likert scale with 53 items with scores ranging from 1 (completely unimportant) to 5 (very important) to represent the importance of each index (each item had an open-ended question to collect the expert's free-text comments); (3) basic characteristics of the experts, including their sex, age, education level, work experience, mentor status, professional title, academic qualifications, and current professional area; and (4) experts' familiarity level and judgment basis relating to the consultation questions.

#### 2.2.2. Recruitment of the Expert Panel

Purposive sampling was used for expert recruitment. Additionally, expert heterogeneity was also considered to obtain a variety of perspectives and achieve more accurate judgments [26], such as different professions (nursing, public health, and hospital infection management), regions, and positions (frontline staff, managers). The selection criteria were as follows: (1) previously participated in the rescue of infectious disease emergencies; (2) ≥10 years of experience in clinical nursing, nursing management, nursing education, public health, or hospital infection management; (3) a bachelor's or higher degree; and (4) willingness to participate in this study. There is no consensus on the necessary number of experts. Belton et al. [26] recommended that 5–20 experts were sufficient; therefore, 18 experts were selected in this study.

#### 2.2.3. Consultation Procedure

The members of the research team contacted the experts, explained the purpose of the research to them, and obtained their consent. The questionnaires were sent to the experts via email. The experts were required to fill out the questionnaires independently and send them back in two weeks. A brief reminder was sent to the nonresponders one day after the deadline.

#### 2.2.4. Data Analysis and Selection of the Index

We used Microsoft Excel 2010 and IBM SPSS Statistics 21.0 to analyze all quantitative data. Frequencies, proportions, and means were used for data descriptions. The authority coefficient of experts was calculated to represent the reliability of the consultation results [27]. The coefficient of variation (CV) was used to judge the coordination and concentration of expert opinions. Kendall's *W* was used to test the consistency of experts' opinions [28]. The qualitative data of experts' free-text comments were analyzed by content analysis [29]. The research team carefully reviewed and discussed the comments from the experts and then modified, excluded, or added items based on the study aims and literature.

After round 1 consultation, the items with a mean importance value <4.5 were deleted. Additionally, the researchers carefully reviewed the experts' comments, categorized the content, integrated similar opinions, and then discussed the research team to modify, exclude, or add items based on the study aims, literature, and theoretical framework of this study. For example, three experts suggested adding items that reflect the “overall view,” “obedient organization arrangements,” and “team spirit” of nurses. Therefore, a related third-level item was added. Two experts suggested changing the second-level index of “communication and management abilities” into “communication abilities.” Through discussion and a literature review, the research team agreed that the comment was reasonable and thus modified the item according to the expert opinion. Finally, several items were modified, and new items were added following analysis of the experts' comments. All the retained items and the new items were entered into the second-round questionnaire. After round 2 of consultation, the items with a mean importance value >4.5 and CV < 0.25 were considered to have reached a consensus [30].

### 2.3. Weight of the Index

We used the analytic hierarchy process to determine the weight of each index. The analytic hierarchy process was completed by Yaahp V10.3 (Meta Decision Software Technology Co., Ltd., Nanjing, China).

#### 2.3.1. Build the Hierarchical Structure Model

In this study, the hierarchical structure model of the index was established according to the result of round 2 of the Delphi consultation, which consisted of four layers: the target layer, the criteria layer, the subcriteria layer, and the scheme layer. In this study, the target layer was the competency assessment index system for nurses responding to infectious disease emergencies, the criteria layer included the 3 first-level indices, the subcriteria layer included the 10 second-level indices, and the scheme layer included the 32 third-level indices.

#### 2.3.2. Construction of the Judgment Matrix

The mean importance value of each index determined by the experts in the round 2 consultation was used to calculate the difference between pairwise indices. Then, the importance degree between pairwise indices was compared, and the pairwise comparison judgment matrix was constructed based on Saaty's fundamental 9-point scale [31]. In this study, we formed a total of 14 judgment matrices.

#### 2.3.3. Test Consistency and Weight Calculation

For consistency testing, a consistency ratio (CR) of the judgment matrix <0.10 indicates that the judgment matrix has satisfactory consistency; in contrast, CR > 0.10 indicates that the judgment matrix needs to be adjusted [30]. In Yaahp V10.3 software, the power method was used to calculate weights.

### 2.4. Ethical Considerations

This study was approved by the Biomedical Research Ethical Committee of West China Hospital, Sichuan University (No. 2020312), and was conducted in accordance with the principles of the Declaration of Helsinki. The study objectives and participant rights were explained to the experts via an electronic document. Consent was implied after the participant completed the survey.

## 3. Results

### 3.1. Characteristics of the Experts

In this study, we conducted two rounds of expert consultation. A total of 18 experts were enrolled from 11 hospitals and universities, covering 7 provinces, autonomous regions, or municipalities, including Hubei, Beijing, Sichuan, Anhui, Gansu, Yunnan, and Xinjiang. The experts' characteristics are shown in Table 1. The response rates of the two rounds of consultations were both 100%. Fifteen (83.33%) and five (27.78%) experts also provided free-text comments in the two rounds. The authority coefficient of the 18 experts was 0.903, indicating that the experts were highly authoritative, and the consultation results were reliable. The concentration and coordination of expert opinions are listed in Table 2. The CVs were 0–0.23 (median 0.13) and 0–0.17 (median 0.09) in the first and second rounds, respectively. Kendall's *W* was 0.170 and 0.139 in the two rounds, with statistically significant differences, indicating a high level of synergy among experts.

### 3.2. Nursing Response Competency Index System of Infectious Disease Emergency

**Figure 1:**
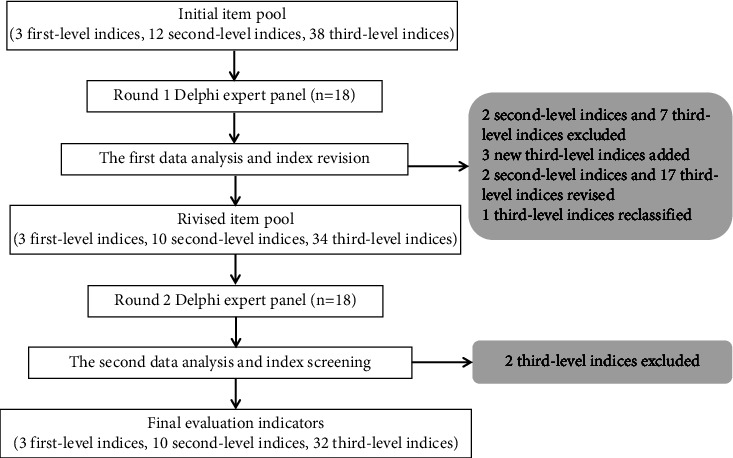
Flowchart of the index construction process. Two rounds of expert consultation were conducted to construct the index system. Through the first round data analysis, 2 second-level indices and 7 third-level indices were deleted, 3 new third-level indices were added, 2 second-level index and 17 third-level index statements were revised, and 1 third-level index was reclassified. Through the second round data analysis, 2 third-level indices were deleted, and 3 third-level indices statements were revised. Finally, the nursing response competency index system of infectious disease emergencies was confirmed, including 3 first-level indices, 10 second-level indices, and 32 third-level indices.

The process of index construction is shown in Figure 1. In the first round, the importance value was 4.11–5.00, with an average of 4.69, and 9 items scored <4.5. In the first round, 62 comments were provided by 15 experts regarding adjusting the wording, merging or splitting some items, and deleting or adding new items. Two second-level indices and 7 third-level indices were deleted, 3 new third-level indices were added, 2 second-level indices, and 17 third-level index statements were revised, and 1 third-level index was reclassified through data analysis. Therefore, a five-point Likert scale with 47 items (3 first-level indices, 10 second-level indices, and 34 third-level indices) was sent to 18 experts for the second round of consultation.

In the second round, the importance value was 4.28–5.00, with an average of 4.78; 2 items scored <4.5, and all CVs were <0.25. The second round also included 13 comments from 5 experts. Two third-level indices were deleted, and 3 third-level index statements were revised. After the second round, the experts' opinions tended to be consistent, and the nursing response competency index system of infectious disease emergencies was confirmed, including 3 first-level indices, 10 second-level indices, and 32 third-level indices (Table 3).

### 3.3. Weight of the Competency Assessment Index

The results showed that the first-level CR was 0.0088, the second-level CR was 0.0825, 0.0000, and 0.0314, and the third-level CR was 0.0266, 0.0000, 0.0000, 0.0000, 0.0000, 0.0192, 0.0172, 0.0000, 0.0000, and 0.0707. All the CRs were <0.1, indicating that each judgment matrix had satisfactory consistency. The weight coefficients of each index are listed in Table 4.

## 4. Discussion

In this study, we developed a competence index system of infectious disease emergency response for nurses in China through a Delphi survey and determined the weight of each index with an analytic hierarchy process. The consensus-based competence index system represents the general expectations of the Chinese health care team for the nurses in response to infectious disease emergencies.

The competence index system established in this study is scientific, reliable, and representative with the following characteristics. First, the response rate of experts indicates their concern for the research, and a response rate >70% is necessary to ensure the rigor of the Delphi technique [32]. The response rate of the two rounds of consultation in this study was 100%, indicating that the experts had high enthusiasm for consultation. Second, a higher degree of expert authority generally indicates better accuracy of prediction, and an authority coefficient ≥0.7 specifies that the consultation results are reliable [27]. In this study, the authority coefficient exceeds 0.9, showing excellent reliability of the experts' opinions. Third, Kendall's *W* in the two rounds was significantly different, indicating good coordination among experts and acceptability of the study results. Additionally, the items' CVs decreased from round 1 to round 2, showing that the experts' opinions tended to be stable and that the consensus was meaningful. Furthermore, the selection of experts is crucial for success in a Delphi study [33]. Purposive sampling was used to improve representativeness, rather than random sampling. In this study, 18 experts were well-known experts in infectious disease nursing, education, or management, and all of them had frontline work experience in infectious disease emergency rescue, such as SARS or COVID-19. They originated from 11 institutions, covering 7 geographic regions, which decreased the regional distribution bias. In summary, the Delphi technique and qualified experts ensure the reliability of this competence index system. Moreover, the combination of the Delphi technique and the analytic hierarchy process method in this study integrates the wisdom of experts and the science of quantitative measurement.

Competency is a complex and holistic concept. Although consensus on the definition and domains of competency is lacking, it contains the integration of knowledge, skills, and attitudes required to accomplish a task effectively and efficiently [18, 20]. Therefore, in this study, the nursing competencies of the infectious disease emergency response initially consisted of 3 first-level indicators—knowledge, attitudes, and skills—and the experts also reached a high consensus. The weights of knowledge, attitudes, and skills were 0.2970, 0.1634, and 0.5396, respectively, reflecting the priority of nursing skills in responding to infectious disease emergencies. Nursing practice is highly technical; therefore, excellent skills are essential for completing basic nursing tasks. In the context of infectious disease emergencies, nurses usually must address many clinical nursing tasks [34]. In this circumstance, nursing skills should be prioritized both in nurse training and in staff deployment. The weight of knowledge ranked second, and most abilities can reasonably be acquired based on knowledge. Finally, although the weight was relatively low, the experts still highly agreed that attitude was indispensable (mean importance value 4.83, CV 0.10). In general, attitude can predict behavior to some extent. The professional attitudes of nurses are essential to enhancing the quality of health care [35]. Professional attitudes are particularly important in response to an infectious disease emergency. Because nurses may be at risk of being infected, stigmatized, isolated, and even sacrificing their lives [36, 37], only nurses with a firm professional attitude and good mental health are suited for frontline nursing work in an infectious disease emergency.

Finally, we confirmed 32 third-level indicators and identified their weight coefficients. These third-level indicators provide evidence for developing training curricula and identifying prioritized training topics in future education. For example, “understand the disinfection and isolation of infectious diseases” (portfolio weight 0.1241) and “correctly perform specimen collection for infectious disease patients” (portfolio weight 0.1245) are the most important knowledge and skills and need to be trained preferentially. Notably, two third-level indicators were also very important. The first indicator was “effectively adjust one's own psychology and behavior to quickly meet the work requirements under the pressure caused by the infectious disease emergency.” The outbreak of an infectious disease and subsequent pandemic exert considerable psychological pressure on health care workers who directly provide care to patients [38]. Studies have demonstrated that nurses who can recover quickly from a stressful event may cope effectively and overcome the pressure imposed by an infectious disease emergency [14]. The second indicator was “follow organization arrangements and collaborate effectively with other team members in an infectious disease emergency.” Good cooperation within a health care team is crucial for providing high-quality care to patients and ensuring the safety and health of staff [34, 39]. In the rescue of an infectious disease emergency, health care workers from various departments, multiple professions, and even different hospitals or institutions may be integrated as a temporary medical aid team to participate in the frontline care work [40]. Team members may not be familiar with each other or with the workflow and the environment in a new workplace; therefore, cooperation on the team is full of challenges. Accordingly, the nurse's team spirit and effective collaboration should be emphasized in this context.

## 5. Limitations

This study was also subjected to limitations. Although the 18 experts recruited nationwide in this study are sufficient for a Delphi study, they may not fully represent all stakeholders. Although purposive sampling was essential to reach the target experts, it may also have introduced bias in the sampling process [30]. Furthermore, the infectious disease emergency response competencies constructed in this study have not yet been tested in a clinical setting. Therefore, the study results should be generalized with caution and limited to a similar context. Future studies need to collect more opinions from various stakeholders to improve the competency index system and test the index system in a real clinical context.

## 6. Conclusion

This study is the first to develop a consensus on infectious disease emergency response competencies needed for nurses in China. A Delphi survey based on a literature review and in-depth expert interviews were used to establish the competency index system, and eventually, 3 first-level indices, 10 second-level indices, and 32 third-level indices were agreed upon. Additionally, the weighted coefficients of each index were determined using the analytic hierarchy process. The competency index system is scientific and reliable, revealing clear expectations and performance standards for nurses in response to an infectious disease emergency and providing guidance for nursing educators to develop effective infectious disease emergency response competency training programs.

## 7. Implications for Nursing Management

The competency index system reveals clear expectations and performance standards for nurses in response to an infectious disease emergency. The findings specifically apply to situations where emergency rescue is required in an infectious disease epidemic or pandemic and the frontline nurses who are deployed to undertake rescue tasks in such an infectious disease emergency. Nursing managers could develop effective training programs for infectious disease emergency response competency for the nurses based on the competency index system and select competent nurses for medical rescue in infectious disease emergencies.

## Figures and Tables

**Table 1:** Characteristics of the experts (*n* = 18).

Variables	*n*	(%)
*Gender*		
Men	2	11.1
Women	16	88.9
*Age (years)*		
≤40	4	22.2
41–50	9	50.0
≥51	5	27.8
*Education level*		
Bachelor degree	4	22.2
Master degree	10	55.6
Doctor degree	4	22.2
*Mentor status*		
Postgraduate tutor	8	44.4
Doctoral supervisor	3	16.7
None	7	38.9
*Working experience(years)*		
10–20	6	33.3
21–30	6	33.3
≥31	6	33.3
*Professional title*		
Nurse supervisor	3	16.7
Associate professor	7	38.9
Professor	8	44.4
*Current professional area*		
Nursing management	5	27.8
Nursing education	2	11.1
Clinical nursing	5	27.8
Public health	1	5.6
Hospital infection management	1	5.6
Two and above	4	22.2

**Table 2:** The level of concentration and coordination of expert's opinion.

Rounds	Importance values	Full-score rates (%)	Coefficients of variation	Kendall's *W*	Chi-square	*P* value
Round1	4.11–5.00	44.00–100	0.00–0.23	0.170	147.565	<0.001
Round2	4.28–5.00	39.00–100	0.00–0.17	0.139	114.780	<0.001

**Table 3:** Consensus of the competency index system after two rounds of the Delphi study.

First-levelindicators	IV^†^	CV^‡^	Second-levelindicators	IV^†^	CV^‡^	Third-level indicators	IV^†^	CV^‡^
(A) Knowledge	4.89	0.06	(A1) General knowledge of infectious disease	4.89	0.06	(A1.1) Understand the epidemic process of common infectious diseases and previous major infectious diseases (source of infection, route of transmission, and susceptible population)	4.89	0.06
						(A1.2) Understand the typical signs and symptoms of common infectious diseases and previous major infectious diseases	4.78	0.11
						(A1.3) Understand the prevention and containment rules of infectious diseases	4.94	0.05
						(A1.4) Understand the treatment and nursing of common and previous major infectious diseases	4.83	0.10
			(A2) Infectious disease management	5.00	0.00	(A2.1) Understand the laws and regulations about infectious disease management	4.72	0.12
						(A2.2) Understand the emergency management procedures related to infectious diseases	4.72	0.12
						(A2.3) Understand the disinfection and isolation of infectious diseases	5.00	0.00
			(A3) Occupational protection	4.61	0.15	(A3.1) Understand the standard precaution	4.89	0.06
						(A3.2) Understand the personal protective requirements of infectious diseases with different transmission routes	5.00	0.00
						(A3.3) Understand the emergency procedures after occupational exposure and medical protective products damage	4.89	0.06

(B) Attitudes	4.83	0.10	(B1) Psychological traits	4.89	0.06	(B1.1) Effectively adjust one's own psychology and behavior to quickly meet the work requirements under the pressure caused by the infectious disease emergency	4.89	0.06
						(B1.2) Have a strong sense of responsibility and dedication in the infectious disease emergency and actively participate in frontline nursing	4.72	0.12
			(B2) Professional attitude	4.83	0.10	(B2.1) Follow organization arrangements and collaborate effectively with other team members in an infectious disease emergency	4.89	0.06
						(B2.2) Provide care for infectious disease patients in accordance with ethics and laws, without stigmatization, fear, or disclosure of patients' privacy	4.78	0.09

(C) Skills	4.94	0.05	(C1) Specialty nursing practice	5.00	0.00	(C1.1) Properly perform respiratory care techniques for patients with infectious disease, e.g., oxygen therapy, atomization inhalation, sputum suction care, endotracheal intubation care, tracheostomy care, and ventilator care	4.61	0.15
						(C1.2) Properly perform circulatory care techniques for patients with infectious disease, e.g., electrocardiographic monitoring and cardiopulmonary resuscitation	4.72	0.12
						(C1.3) Identify anticipated condition changes and provide effective care for patients with infectious disease	4.67	0.12
						(C1.4) Identify the psychological needs of infectious disease patients and their families and give appropriate support	4.67	0.10
						(C1.5) Correctly perform specimens collection for infectious disease patients	4.83	0.08
			(C2) Hospital infection management	4.67	0.12	(C2.1) Comply with the quarantine requirements of different areas (clean, potential contamination, and/or contamination area) and channels	5.00	0.00
						(C2.2) Correctly classify and provide quarantine measures for infectious disease patients, e.g., placement of patients, selection of medical protective products, and management of visiting and accompanying	4.89	0.06
						(C2.3) Correctly perform disinfection and dispose of the environment, belongings, cadavers, medical waste, and medical reusable materials of infectious disease patients	4.89	0.06
						(C2.4) Put on and take off personal protective equipment according to the quarantine type and the protection level (level 1 protection, level 2 protection, and level 3 protection)	4.89	0.06
						(C2.5) Properly perform hand hygiene	4.83	0.08
						(C2.6) Manage the personnel and environment in the ward to reduce the risk of infectious diseases spread	4.72	0.09
			(C3) Education and consulting	4.50	0.13	(C3.1) Effectively teach the prevention, containment, and rehabilitation measures about infectious disease to patients and their families	4.72	0.09
						(C3.2) Guide other health team members in caring for patients with infectious diseases	4.72	0.09
			(C4) Communication abilities	4.72	0.09	(C4.1) Effectively communicate with infectious disease patients and their families	4.67	0.12
						(C4.2) Effectively communicate and coordinate with health team members during infectious disease emergencies	4.83	0.08
			(C5) Thinking and learning abilities	4.67	0.10	(C5.1) Under the pressure of infectious disease emergency, remain critical thinking and make correct decisions to solve difficulties in work	4.56	0.13
						(C5.2) Identify and deal with potential risks related to infectious disease emergency response	4.67	0.10
						(C5.3) Actively and effectively learn new knowledge and skills to improve the required abilities for infectious diseases patient care	4.83	0.10

*Note*. ^†^IV: importance value; ^‡^CV: coefficient of variation.

**Table 4:** The weights coefficient of each indicator.

First-levelindicators	Weights	Second-level indicators	Weights	Third-level indicators	Weights	Portfolio weights
(A) Knowledge	0.2970	(A1) General knowledge of infectious disease	0.2797	(A1.1) Understand the epidemic process of common infectious diseases and previous major infectious diseases (source of infection, route of transmission, and susceptible population)	0.2829	0.0235
				(A1.2) Understand the typical signs and symptoms of common infectious diseases and previous major infectious diseases	0.1059	0.0094
				(A1.3) Understand the prevention and containment rules of infectious diseases	0.4476	0.0396
				(A1.4) Understand the treatment and nursing of common and previous major infectious diseases	0.1636	0.0145
		(A2) Infectious disease management	0.6267	(A2.1) Understand the laws and regulations about infectious disease management	0.1667	0.0310
				(A2.2) Understand the emergency management procedures related to infectious diseases	0.1667	0.0310
				(A2.3) Understand the disinfection and isolation of infectious diseases	0.6667	0.1241
		(A3) Occupational protection	0.0936	(A3.1) Understand the standard precaution	0.2500	0.0069
				(A3.2) Understand the personal protective requirements of infectious diseases with different transmission routes	0.5000	0.0139
				(A3.3) Understand the emergency procedures after occupational exposure and medical protective products damage	0.2500	0.0069

(B) Attitudes	0.1634	(B1) Psychological traits	0.6667	(B1.1) Effectively adjust one's own psychology and behavior to quickly meet the work requirements under the pressure caused by the infectious disease emergency	0.7500	0.0817
				(B1.2) Have a strong sense of responsibility and dedication in the infectious disease emergency and actively participate in frontline nursing	0.2500	0.0274
		(B2) Professional attitude	0.3333	(B2.1) Follow organization arrangements and collaborate effectively with other team members in an infectious disease emergency	0.7500	0.0822
				(B2.2) Provide care for infectious disease patients in accordance with ethics and laws, without stigmatization, fear, or disclosure of patients' privacy	0.2500	0.0274

(C) Skills	0.5396	(C1) Specialty in nursing practice	0.5352	(C1.1) Properly perform respiratory care techniques for patients with infectious disease, e.g., oxygen therapy, atomization inhalation, sputum suction care, endotracheal intubation care, tracheostomy care, and ventilator care	0.0773	0.0223
				(C1.2) Properly perform circulatory care techniques for patients with infectious disease, e.g., electrocardiographic monitoring and cardiopulmonary resuscitation	0.2224	0.0642
				(C1.3) Identify anticipated condition changes and provide effective care for patients with infectious disease	0.1330	0.0384
				(C1.4) Identify the psychological needs of infectious disease patients and their families and give appropriate support	0.1330	0.0384
				(C1.5) Correctly perform specimens collection for infectious disease patients	0.4343	0.1254
		(C2) Hospital infection management	0.1163	(C2.1) Comply with the quarantine requirements of different areas (clean, potential contamination, and/or contamination area) and channels	0.3772	0.0237
				(C2.2) Correctly classify and provide quarantine measures for infectious disease patients, e.g., placement of patients, selection of medical protective products, and management of visiting and accompanying	0.1570	0.0099
				(C2.3) Correctly perform disinfection and dispose of the environment, belongings, cadavers, medical waste, and medical reusable materials of infectious disease patients	0.1570	0.0099
				(C2.4) Put on and take off personal protective equipment according to the quarantine type and the protection level (level 1 protection, level 2 protection, and level 3 protection)	0.1570	0.0099
				(C2.5) Properly perform hand hygiene	0.0936	0.0059
				(C2.6) Manage the personnel and environment in the ward to reduce the risk of infectious diseases spread	0.0583	0.0037
		(C3) Education and consulting	0.0547	(C3.1) Effectively teach the prevention, containment, and rehabilitation measures about infectious disease to patients and their families	0.5000	0.0148
				(C3.2) Guide other health team members in caring for patients with infectious diseases	0.5000	0.0148
		(C4) Communication abilities	0.1697	(C4.1) Effectively communicate with infectious disease patients and their families	0.2500	0.0229
				(C4.2) Effectively communicate and coordinate with health team members during infectious disease emergencies	0.7500	0.0687
		(C5) Thinking and learning abilities	0.1240	(C5.1) Under the pressure of infectious disease emergency, remain critical thinking and make correct decisions to solve difficulties in work	0.1172	0.0078
				(C5.2) Identify and deal with potential risks related to infectious disease emergency response	0.2684	0.0180
				(C5.3) Actively and effectively learn new knowledge and skills to improve the required abilities for infectious diseases patient care	0.6144	0.0411

*Note*. The first-level consistency ratio (CR) was 0.0088; the second-level CR was 0.0825, 0.0000, and 0.0314, respectively; and the third-level CR was 0.0266, 0.0000, 0.0000, 0.0000, 0.0000, 0.0192, 0.0172, 0.0000, 0.0000, and 0.0707, respectively.

## Data Availability

The data analyzed during the current study are not publicly available due to privacy restrictions but are available from the corresponding authors on reasonable request.
